# A dual-crosslinking electroactive hydrogel based on gelatin methacrylate and dibenzaldehyde-terminated telechelic polyethylene glycol for 3D bio-printing

**DOI:** 10.1038/s41598-024-54853-9

**Published:** 2024-02-19

**Authors:** Yulong Wang, Songsong Yang, Heqing Cai, Hailong Hu, Kun Hu, Zhicheng Sun, Ruping Liu, Yen Wei, Lu Han

**Affiliations:** 1 The Engineering Research Center of 3D Printing and Bio-Fabrication, Beijing Institute of Graphic Communication, Beijing, 102600 China; 2 School of Physical Science and Technology, Guangxi University, Nanning, 530004 China; 3 The Key Laboratory of Bioorganic Phosphorus Chemistry and Chemical Biology (Ministry of Education), Department of Chemistry, Tsinghua University, Beijing, 100084 China

**Keywords:** Biological techniques, Biotechnology, Materials science

## Abstract

Gelatin was widely used as scaffold materials in 3D bio-printing due to its excellent bioactivity and availability and especially that their arginine–glycine–aspartic acid (RGD) sequences could efficiently promote cell adhesion and proliferation. In this study, an electroactive and 3D bio-printable hydrogel was prepared through a two-step chemical cross-linking process. Specifically, residual free amino groups of methacrylated gelatin (GelMA) were cross-linked with the aldehyde groups of dibenzaldehyde-terminated telechelic polyethylene glycol (DF-PEG) via Schiff base bonds, forming a gel at 37 °C. During the subsequent 3D bio-printing process, GelMA underwent UV curing, forming a secondary cross-linked network to the mechanical strength and stability of the printed structure. The uniform dispersion of carbon nanotubes (CNTs) in the GelMA/DF-PEG composite hydrogel significantly increased its conductivity. The optimized GelMA/DF-PEG composite hydrogel, i.e., 30% GelMA and 25% DF-PEG (G30D25-CNTs), exhibited superior bio-printability. When the content of CNTs was above 4%, the conductivity of G30D25-CNTs hydrogel exceeded 10^–2^ S/m, which satisfied the needs of cells for micro-current stimulation. Furthermore, the pore microstructures, swelling behavior, degradation ability and cell toxicity of G30D25-CNTs electroactive hydrogels were thoroughly evaluated. Thus, the G30D25-CNTs hydrogel with 4% MWCNTs could be considered for further application in electrical stimulation of tissue regeneration such as muscle and cardiac nerve tissue repair.

## Introduction

3D bio-printing technology has significantly improved the capability to fabricate artificial tissues or organs via layer-by-layer stacking of biomaterials and cells^1–3^. However, a vital yet limiting aspect is the lack of scaffold materials^4,5^. Hydrogel is an ideal candidate used as tissue engineering scaffold, which have the following three properties: (i) certain mechanical strength to maintain printed 3D structure. (ii) Shear thinning property. Hydrogel could be smoothly extruded through the nozzle and then maintain the shape fidelity of 3D printed structure. (iii) Excellent biocompatibility, which can promote cell adhesion and proliferation^6–8^.

Gelatin was usually chosen as a kind of scaffold materials due to its ready-availability and its excellent bioactivity^9–11^. More importantly, there are arginine–glycine–aspartic acid (RGD) sequences on gelatin molecule, which promotes cell adhesion and proliferation without addition of external cell adhesive ligands^12–14^. Therefore, gelatin-based hydrogels are ideal tissue engineering scaffold materials. Normally, physically cross-linked methods were employed to prepare gelatin-based hydrogels such as gelatin/alginate^15,16^, gelatin/hyaluronic acid^17,18^ and gelatin/fibrin^19^, the mechanical properties of which are extremely weak. When 3D printed constructs using the above gelatin-based hydrogels are immersed into culture medium at 37 °C, they collapse immediately due to their special thermo-responsive property^13^. To improve its thermal stability of gelatin, various approaches have been explored. Some chemical agents, such as aldehydes (e.g., glutaraldehyde or glyceraldehyde), genipin, polyepoxides and isocyanates, have been utilized to cross-link the gelatin-based hydrogels^20,21^. Nevertheless, these chemical cross-linking agents are toxic to cells. Another effective approach is to modify gelatin with a methacrylamide group i.e., GelMA^22–24^, which can form a thermo-irreversible hydrogel under ultraviolet irradiation. Furthermore, composite hydrogels can be obtained by blending GelMA with other polymers, which possess much higher viscosity than GelMA alone. Moreover, GelMA-based hydrogels frequently demonstrate inadequate conductivity, rendering them unsuitable for meeting the electrical stimulation criteria necessary for muscle and neural tissues. Carbon nanotubes (CNTs) are widely employed to improve their conductivity, mechanical characteristics, and biocompatibility^25–28^. Shin et al.^29^ have pioneered the development of gelatin CNTs/GelMA hydrogel films, which have been shown to enhance the structural organization and functionality of cardiomyocytes. Nonetheless, the exploration of CNTs/GelMA composite hydrogels with exceptional 3D bio-printing capabilities continues to present a significant challenge.

Polyethylene glycol (PEG) is an FDA-approved polymer, which has the advantages of hydrophilicity, non-toxicity, biocompatibility and non-immunogenicity. Moreover, there are two hydroxyl groups at each end, which can be easily modified with other functional groups^30,31^. Therefore, it is often used for surface modification, bio-conjugation, drug delivery and tissue engineering^32^. In this work, two hydroxyl groups of PEG were firstly modified to obtain a dibenzaldehyde-terminated telechelic PEG. Then, the composite hydrogels were obtained through schiff base bonds between the aldehyde groups of the difunctionalized polyethylene glycol (DF-PEG) and the amino groups of GelMA. Furthermore, a certain amount of multi-walled carbon nanotubes (MWCNTs) was uniformly dispersed to obtain the GelMA/DF-PEG-CNTs electroactive hydrogel, which underwent UV curing during the subsequent 3D bio-printing process to improve the mechanical strength and stability of the printed structure. Finally, swelling property, degradability and conductivity of the electroactive hydrogel were thoroughly examined.

## Experimental

### Materials

Gelatin (300 KDa, Type A, from porcine skin) was purchased from VETEC. Polyethylene glycol (PEG) (2000 KDa), 4-(dimethylamino) pyridine (DMAP), methacrylic anhydrideand and Irgacure 2959 were obtained from Sigma-Aldrich (St. Louis., MO, USA). 4-formylbenzoic acid (AR, 98%), N, Nʹ-dicyclohexylcarbodiimide (DCC) and 2, 4, 6-trinitrobenzenesulfonic acid (TNBS) were purchased from Aladdin Reagent Company (Shanghai, China). Multi-walled carbon nanotubes (MW-CNTs) were purchased from Chengdu Organic Chemicals Co. Ltd., Chinese Academy of Sciences. Dulbecco’s Modified Eagle’s Medium (DMEM), CCK-8 Cell Proliferation and Cytotoxicity Assay Kit, and Calcein-AM/PI Assay Kit were acquired from Beijing Solarbio Science & Technology Ltd (China). MC3T3-E1 Cell was purchased from American Type Culture Collection (Manassas, VA). Tetrahydrofuran (THF) was distilled prior to use, and other reagents were of analytical grade and without further purification.

### Synthesis of dibenzaldehyde-functionalized PEG (DF-PEG)

DF-PEG was prepared by esterification of PEG2000 with 4-formylbenzoic acid^33^. Briefly, 4-formylbenzoic acid (0.602 g, 4.0 mM), PEG2000 (2.0 g, 1.0 mM) and DMAP (0.05 g) were dissolved in 50 ml of dry THF, followed by the addition of DCC (0.825 g, 4.0 mM) under a nitrogen atmosphere, and the system was stirred for 20 h at 25 °C. Then, the white solid was filtered out from the turbid solution. DF-PEG was obtained as a white solid after repeated dissolution in THF and precipitation in diethyl ether for three times. Upon drying, 2.03 g of DF-PEG was obtained at 78% yield. 1H NMR spectra of the DF-PEG was recorded on a Bruker Avance 300 MHz spectrometer. Multiplicities were reported as singlet (s), doublet (d), triplet (t), and multiplet (m). FT-IR analyses were carried out using a Nicolet 6700 Fourier transform infrared spectrometer.

### Preparation of GelMA/DF-PEG hydrogel

First, gelatin methacrylate (GelMA) was prepared by chemically modified the Ɛ-amino groups on gelatin with methacrylic anhydride as previously reported method^9,34^. In this process, certain amount of methacrylic anhydride was dropwise added into gelatin aqueous solution to obtain partially amidated gelatin. TNBS was utilized as an ultraviolet chromophore to assess the level of free amine groups in GelMA samples, as the methacrylate groups of TNBS specifically bound to free amine groups^35^. In brief, 100, 200, 300, 400 and 500 μl of 1 mg/ml GelMA were dispensed into centrifugal tubes and adjusted to a volume of 1 ml. 1 ml of pH8.5 NaHCO3 and 1 ml of 0.1% TNBS solution were added, and the mixture was allowed to react at 40°C for 2 h. Following this, 0.5 ml of 1 M HCl was added, and the absorbance at a wavelength of 346 nm was measured using a UV spectrometer. Pure gelatin solution was used as the control group. The absorption and concentration curves were drawn, and the curves of gelatin and modified gelatin were linearly fitted to obtain the linear slope. The degree of modification of GelMA was calculated using the formula:

$$Degree \left(\%\right)=1-\left(\frac{{k}_{x}}{{k}_{0}}\right)\times 100\%,$$

where ${k}_{x}$ and ${k}_{0}$ represent the slope of modified GelMA and gelatin, respectively”.

Subsequently, 30% GelMA solution (w/v) was prepared by dissolving lyophilized GelMA with 30% degree of methacrylation in PBS containing 0.25% (w/w) Irgacure 2959. MW-CNTs were then added into GelMA solution and stirred for another 12 h. Finally, DF-PEG was added, and the hydrogel formed immediately. For different addition of MW-CNTs, the GelMA/DF-PEG/MW-CNTs hydrogels were denoted as G30D25-CNTs 0.5, G30D25-CNTs 1, G30D25-CNTs 2, G30D25-CNTs 4 and G30D25-CNTs 6, respectively. Finally, the G30D25-CNTs hydrogels were subjected to a 365 nm wavelength UV lamp (10 mW/cm^2^) to trigger secondary chemical crosslinking during the 3D bio-printing process.

### Rheological and mechanical properties of G30D25-CNTs hydrogel

The rheological properties of GelMA/DF-PEG with various MW-CNTs contents were measured using a plate rheometer (TA2000ex, TA Instruments, Inc.). As a typical operation, hydrogel samples were spread on the sample stage, and then the viscosities with changing the shear rates as well as the changes of the storage modulus Gʹ and the loss modulus G" with varying the frequency were measured at 37 °C. The shear rate and the angular frequency were set from 0.01 to 100 s^−1^ and 0.1 to 100 rad^−1^, respectively.

For mechanical testing, G30D25-CNTs samples were transferred into a 20 ml syringe and then exposed to 365 nm UV light (10 mW/cm^2^) for 5 min. An electronic universal tensile testing machine (YL-S90, Yuelian Testing Machines Co., Ltd, Dongguan, China) was used to perform the compression test of the hydrogels.

### Morphological characterization

The morphologies of GelMA/DF-PEG, and GelMA/DF-PEG/MW-CNTs hydrogels were observed under a scanning electron microscope (SEM, SU8020, Japan). The hydrogel samples were prefrozen in a refrigerator at − 20 °C for 2 h and then freeze-dried for 20 h to prepare SEM samples. The test samples were sputter-coated with a gold layer and followed by the experiment was performed at an accelerating voltage of 5 kV.

### Electroactive scaffolds fabricated by 3D bioprinting

The prepared GelMA/DF-PEG and GelMA/DF-PEG/MW-CNTs hydrogels were fed into a syringe and were fixed in the barrel of 3D-Bioplotter (Envision TEC, Germany). The temperature of the syringe barrel was maintained at 37 °C using a temperature controller. The hydrogels were dispensed through a metal needle (nozzle diameter, 22G, 0.6 mm). The deposition speed and pressure were controlled by customized software developed by 3D-Bioplotter. For G30D25, G30D25-CNTs 0.5, G30D25-CNTs 1, G30D25-CNTs 2, G30D25-CNTs 4 and G30D25-CNTs 6 samples, the optimum pressure was 2.0, 2.1, 2.3, 2.5, 2.7 and 2.8 bar, correspondingly, and the deposition speed was 10, 9, 8, 8, 10 and 9 mm/s, correspondingly. Subsequent layers were deposited at a 90° angle to the underlying layer. At the end, the mechanical strength and shape fidelity of printed scaffolds were further improved by triggering the secondary chemical crosslinking of intramolecular GelMA through exposure to 365 nm UV light (10 mW/cm^2^).

### Conductivity measurement

The conductivities of prepared hydrogels were measured using a 3D-printing apparatus as we reported previously^36^. Briefly, the printed scaffolds using prepared hydrogels were sandwiched between a pair of copper plate. And then four screws at corners of the 3D-printed measuring apparatus were tightened to assure that printed scaffolds could closely contact with the copper plates. Two external conducting wires were linked to a PGSTAT302N electrochemical workstation via the reserved grooves.

Electrochemical impedance spectroscopy (EIS) of GelMA/DF-PEG and GelMA/DF-PEG/MW-CNTs hydrogels was acquired and then simulated using Zsimpwin modeling software to obtain the bulk resistance of these hydrogels (Rgel). The Rgel values were finally converted into conductivity (κ) according to the following equation: κ = L/(S × Rgel), where L and S represent the distance between electrodes and the area of electrode contacting with printed scaffolds, respectively.

### Wettability, swelling and degradation behavior of G30D25-CNTs hydrogel

The wettability of the G30D25-CNTs hydrogels was measured using water contact angle analysis by the contact angle tester (XG-CAM). 10 μl of DI water was dropped on to the surface of G30D25-CNTs and an image of the droplet was recorded. Contact angles were recorded in three different areas of each specimen (n = 3).

All the hydrogel samples (10 mm in diameter, 4 mm in height) were freeze-dried to a constant weight and then immersed in PBS (pH 7.4) at 37 °C. At multiple time points, the swollen hydrogels were taking out and the excess of water on surface was absorbed using filter paper, and then the swollen hydrogels were weighed. The swelling ratio (*SR*) was calculated as follows:

1 $$SR=(Ws-Wd)/Wd,$$

where *Ws* and *Wd* represent the weights of hydrogel samples in swelling state and in dry state, respectively. Data from each sample was calculated using triplicate measurement.

The hydrogels were weighted after freeze-drying and then placed in a dry tea bag, which was then immersed in PBS supplemented with NaN3 and kept at 37 °C. At definite time intervals, the samples were dabbed dry and weighed. The relative percentage of degradation (*Wr*) was calculated by *Wr* = (*W*1*/W*0) × 100%, where *W*0 and *W*1 are the weights of a hydrogel sample before and after soaking, respectively. Data from each sample was calculated using triplicate measurement.

### In vitro cytotoxicity analysis

To assess the in vitro biocompatibility of G30D25-CNTs hydrogels, CCK-8 Cell Proliferation and Cytotoxicity Assay Kits were used. Briefly, a suspension of mouse embryo osteoblast precursor cells (MC3T3-E1) was seeded in 96-well plates at a density of 8 × 10^3^ cells (100 μl/well). After overnight incubation, the seeded cells are fully attached. Then, the cells were co-cultured with extracting liquid from G30D25-CNTs hydrogels in a cell incubator (at 37 °C, 5% CO2). After 24 h, the DMEM medium was removed and fresh DMEM medium was added to the 96-well plates. Then CCK-8 solution (10 μl/well) was carefully added dropwise and incubated for 1 h. Finally, the absorbance at 450 nm was measured using a microplate reader.

A Calcein-AM/PI Staining Reagent was used to stain live/dead cells based on green fluorescence imaging of calcein-AM and red fluorescence imaging of PI. MC3T3-E1 cells (1 × 10^5^ cells per well) were incubated in 6-well culture plates with 2 ml DMEM medium (containing 0.1 mg/ml extracting liquid) for 24 h co-culture. Following this, the medium was removed and MC3T3-E1 cells were stained by adding the mixture of 10 μg/ml Calcein-AM and 15 μg/ml PI in PBS for 30 min. Finally, the mixture was removed and washed with PBS three times. An inverted fluorescence microscope (Leica DMI16000B, Germany) was then used to collect the Live/Dead fluorescent signal (AM λex = 490 nm, PI λex = 535 nm).

## Results and discussion

### Fabrication of GelMA/DF-PEG hydrogel

**Figure 1:**
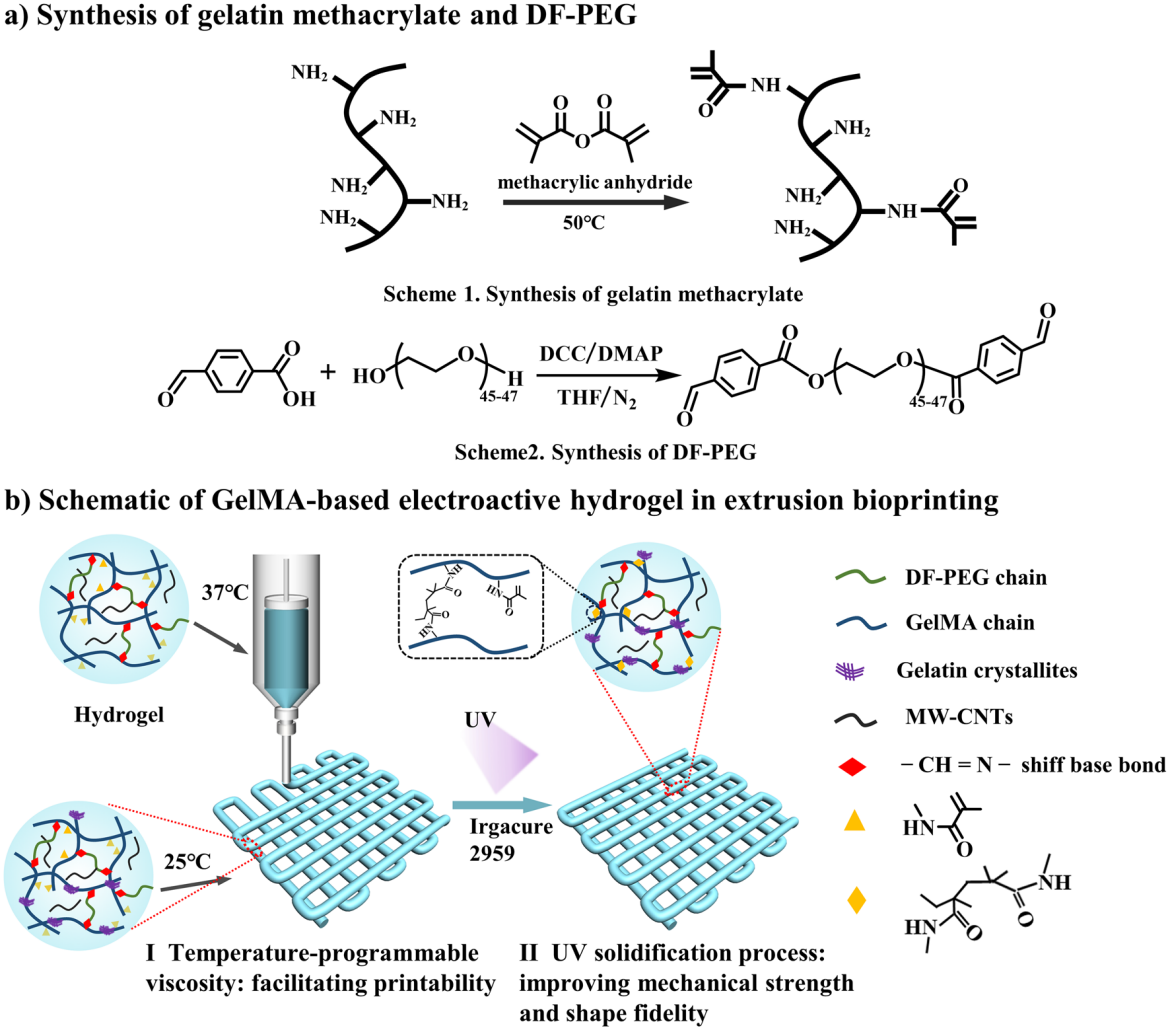
Preparation and utilization of novel gelatin methacrylate-based hydrogel. (**a**) Schematic showing the synthetic steps of the two molecules required for the preparation of the bioprinted gel. (**b**) Schematic illustration of GelMA-based hydrogel during extrusion bioprinting.

The preparation of the GelMA/DF-PEG hydrogel was mainly completed in three steps. First, partially amidated gelatin and double-end aldehyde group-modified polyethylene glycol were synthesized as shown in Schemes 1 and 2 in Fig. 1a, respectively. Subsequently, the aqueous solutions of two polymers were mixed under constant stirring at 37 °C, and the hydrogel was rapidly formed due to the Schiff-base reaction between the residual amino group of GelMA and the aldehyde group of the linear crosslinker DF-PEG.

**Figure 2:**
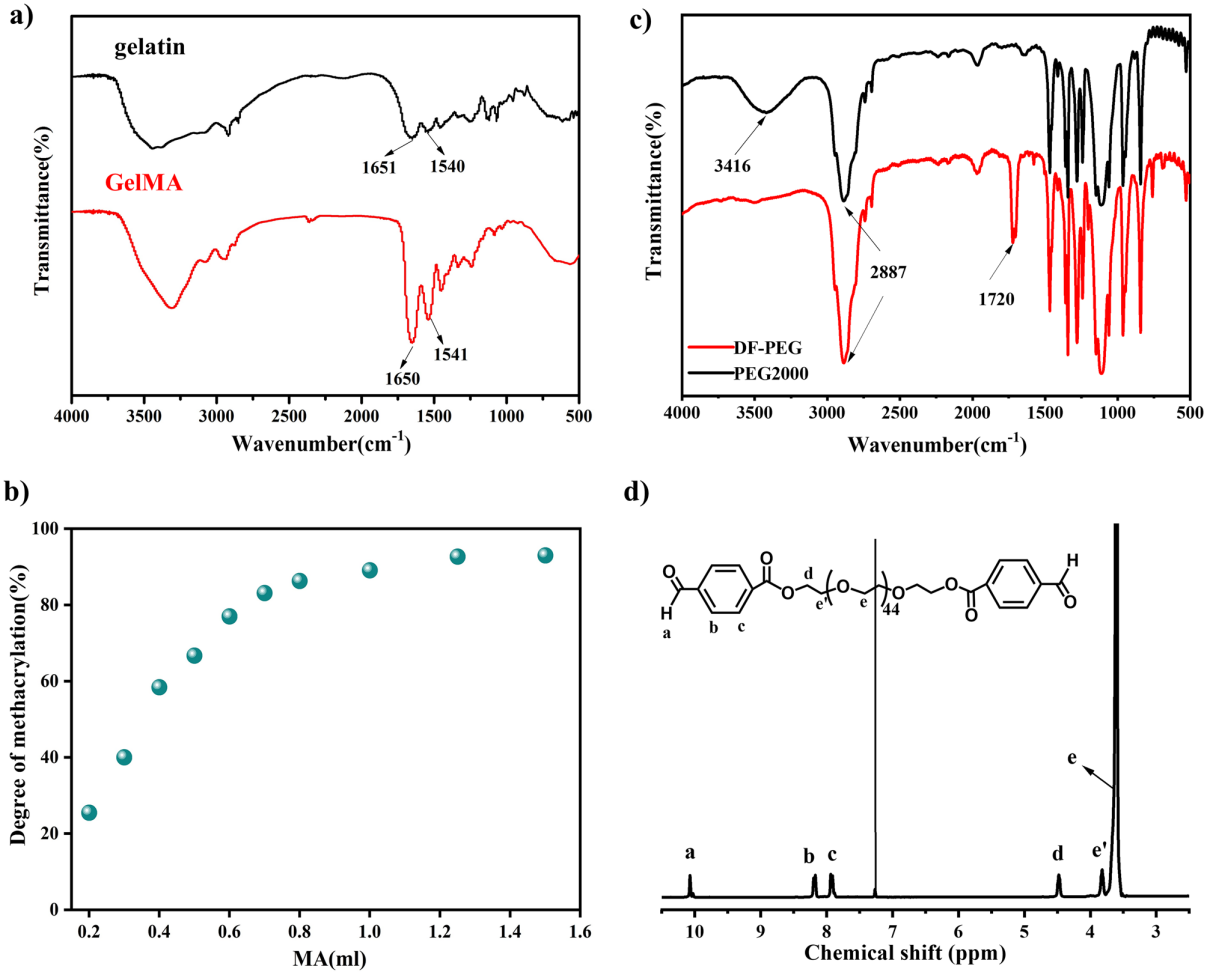
Characterization of GelMA and DF-PEG (**a**) FT-IR spectra of gelatin and GelMA. (**b**) Variation of the degree of gelatin methacrylate with the addition of MA, (**c**) FT-IR spectra of DF-PEG and PEG_2000_ and (**d**) ^1^H NMR spectrum of DF-PEG in CDCl_3_.

The infrared spectra of gelatin and gelatin methacrylate (GelMA) were shown in Fig. 2a. The characteristic peaks at 1651 cm^−1^ and 1540 cm^−1^ represent amide I (C=O stretching vibration) and amide II (C–N stretching vibration and N–H stretching vibration) in gelatin molecules, respectively. Meanwhile, it can be seen from the spectrum of GelMA that the peak of amide I at 1650 cm−^-1^ and the peak of amide II at 1541 cm^−1^ are significantly enhanced, and no new characteristic peaks appeared. This result was consistent with literature reports^37^, indicating that the GelMA was successfully prepared. Under sufficient reaction time, the degree of methacrylation of gelatin was determined by the amount of methacrylic anhydride added, and the detailed results were shown in Fig. 2b. GelMA with a high degree of methacrylate substitution was inconducive to the formation of G30D25 gelation via the Schiff base bonds. Therefore, GelMA with 30% degree of methacrylation was selected for subsequent experiments.

DF-PEG was prepared by esterification of hydroxylterminated PEG with 4-formylbenzoic acid as shown in Scheme 2 in Fig. 1. In the FT-IR spectrum of DF-PEG (Fig. 2c), the original hydroxyl peak at 3416 cm^−1^ almost disappeared. The new aldehyde and ester carbonyls peaks could also be clearly identified at 1720 cm^−1^ and 1712 cm^−1^, respectively. In 1H NMR (300 MHz, CDCl3, δ) of DF-PEG (Fig. 2d): a, 10.07 (s, 2H, CHO); b, 8.23 (d, 4H, CHCCHO); c, 7.85 (d, 4H, CHCHCCHO); d, 4.49–4.44 (m, 4H, COOCH2); eʹ, 3.88–3.84 (m, 4H, COOCH2CH2); e, 3.67–3.56 (m, 172-176H, OCH2CH2O). The protons of methylene ether gave signals at 3.67–3.56 ppm. The aldehyde (10.07 ppm), benzene ring (8.23, 7.85 ppm) and ester methylene (4.49–4.44 ppm) groups were clearly observed. This result indicated that, PEG polymer was successfully terminated with benzaldehyde groups at both ends.

### Rheological property of G30D25-CNTs hydrogels

**Figure 3:**
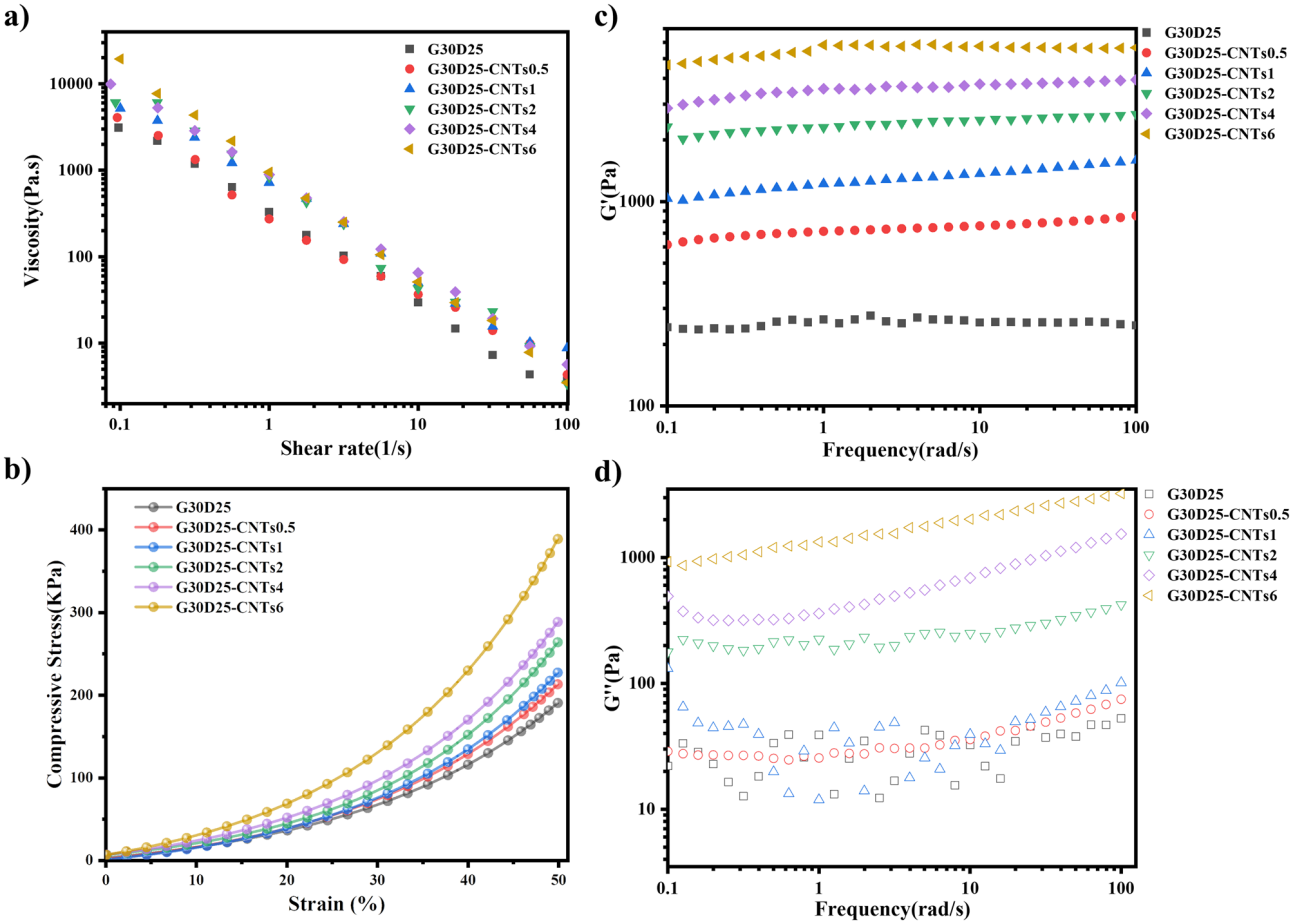
Rheological and mechanical properties of G30D25 and G30D25-CNTs hydrogels. (**a**) Shear viscosity as a function of shear rate at 37 °C. (**b**) Compressive stress–strain curve at up to 50% strain, (**c**) the storage modulus (Gʹ) and (**d**) the loss modulus (Gʹʹ) of the composite hydrogels at 37 °C.

The rheological property of hydrogels was essentially important for extrusion-based 3D bioprinting^6^. As shown in Fig. 3a, the viscosity of G30D25 and G30D25-CNTs hydrogels decreased with increasing the shear rate, indicating their shear-thinning behavior. These hydrogels have relatively low viscosity at high shear rate and much higher viscosity at low shear rate, therefore they can be easily extruded and retained its plotted shape once being printed via 3D bioprinter^38,39^. It was also observed that, G30D25-CNTs hydrogels exhibited a comparatively higher viscosity than G30D25 alone, which was attributed to the entanglements between G30D25 and CNTs.

The mechanical strength of hydrogels is also a crucial consideration as a scaffold material. The compressive stress–strain curve of the G30D25-CNTs hydrogel is presented in Fig. 3b. We can see that all the G30D25-CNTs hydrogel exhibit an initial rigid response. With increasing strain, the internal molecular chains of the G30D25-CNTs hydrogel undergo compression, leading to a continuous increase in compressive strength. Moreover, as the CNTs content increases, offering more rigid structures and non-covalent interactions, the mechanical performance of the G30D25-CNTs hydrogel is enhanced, resulting in an increase of compressive strength.

The storage modulus (Gʹ) and the loss modulus (Gʹʹ) of G30D25 and G30D25-CNTs hydrogels at 37 °C were shown in Fig. 3c,d, respectively. It was evident that the storage modulus Gʹ shows almost no dependence with frequency. G30D25 and G30D25-CNTs hydrogels all demonstrated elastic behavior (Gʹ > Gʹʹ) at detected frequencies, indicating that they all behave typically as fully elastic gels^40^. Moreover, the storage moduli of G30D25-CNTs were all higher than that of G30D25. Benefiting from the high mechanical properties of CNTs, as the concentration of CNTs increased, the Gʹ values of G30D25-CNTs is enhanced accordingly, illustrating that the viscosity and mechanical properties of G30D25-CNTs were improved. These excellent rheological properties can help to maintain the layer-by-layer structure printed with G30D25-CNTs composite hydrogels without any collapsing situation.

### Morphology of G30D25-CNTs hydrogles

**Figure 4:**
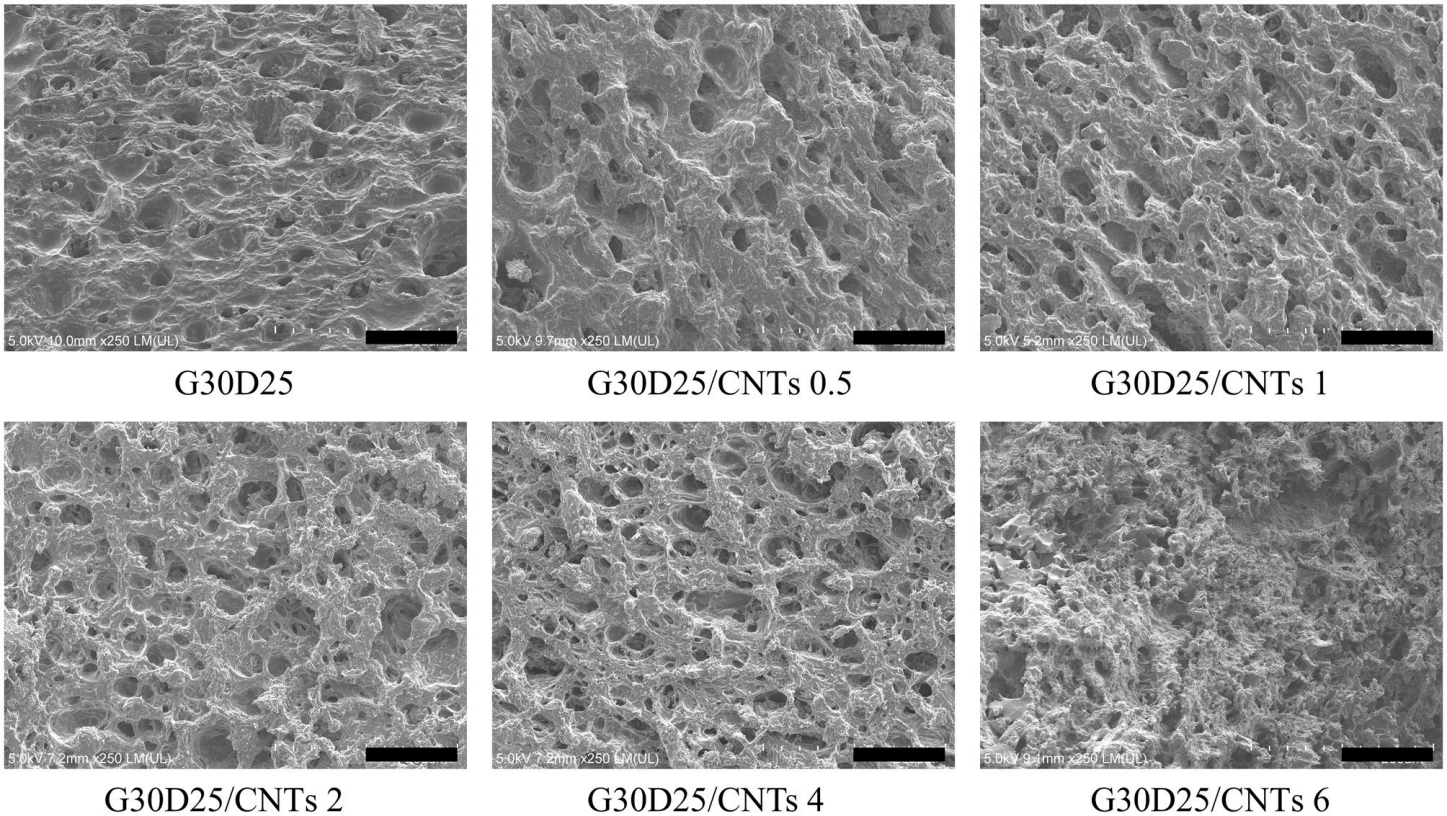
The microscopic morphology of G30D25-CNTs hydrogels, scale bar is 100 μm.

The porous microstructure of tissue engineering scaffolds had significant influence on the ability of cell adhesion, proliferation and differentiation^41^. Therefore, as a material for tissue engineering scaffolds, it must first possess a highly porous structure, with interconnected pores, so that cells can grow throughout the scaffold^42^. The SEM images of G30D25-CNTs hydrogels (Fig. 4) reveal that the G30D25 and G30D25-CNTs0.5 groups exhibit very few interconnected pores, which is not conducive to uniform cell growth throughout the scaffold. In G30D25-CNTs6, the addition of too many CNTs results in the smallest pore size. In contrast, when the concentration of CNTs was raised from 1 to 4%, G30D25-CNTs1, G30D25-CNTs2, and G30D25-CNTs4 exhibit significantly more interconnected pores microstructures with higher porosity, so that cells can grow throughout the scaffolds. The pore sizes of G30D25-CNTs1, G30D25-CNTs2 and G30D25-CNTs4 were 16.57 ± 8.79, 16.87 ± 10.87 and 15.29 ± 9.89 μm, respectively (Fig. S1), which allows cell migration and the delivery and diffusion of vital cell nutrients. This result implied that G30D25-CNTs1, G30D25-CNTs2 and G30D25-CNTs4 were more suitably used as tissue engineering scaffolds^42^.

### 3D bioprinting G30D25-CNTs scaffolds

**Figure 5:**
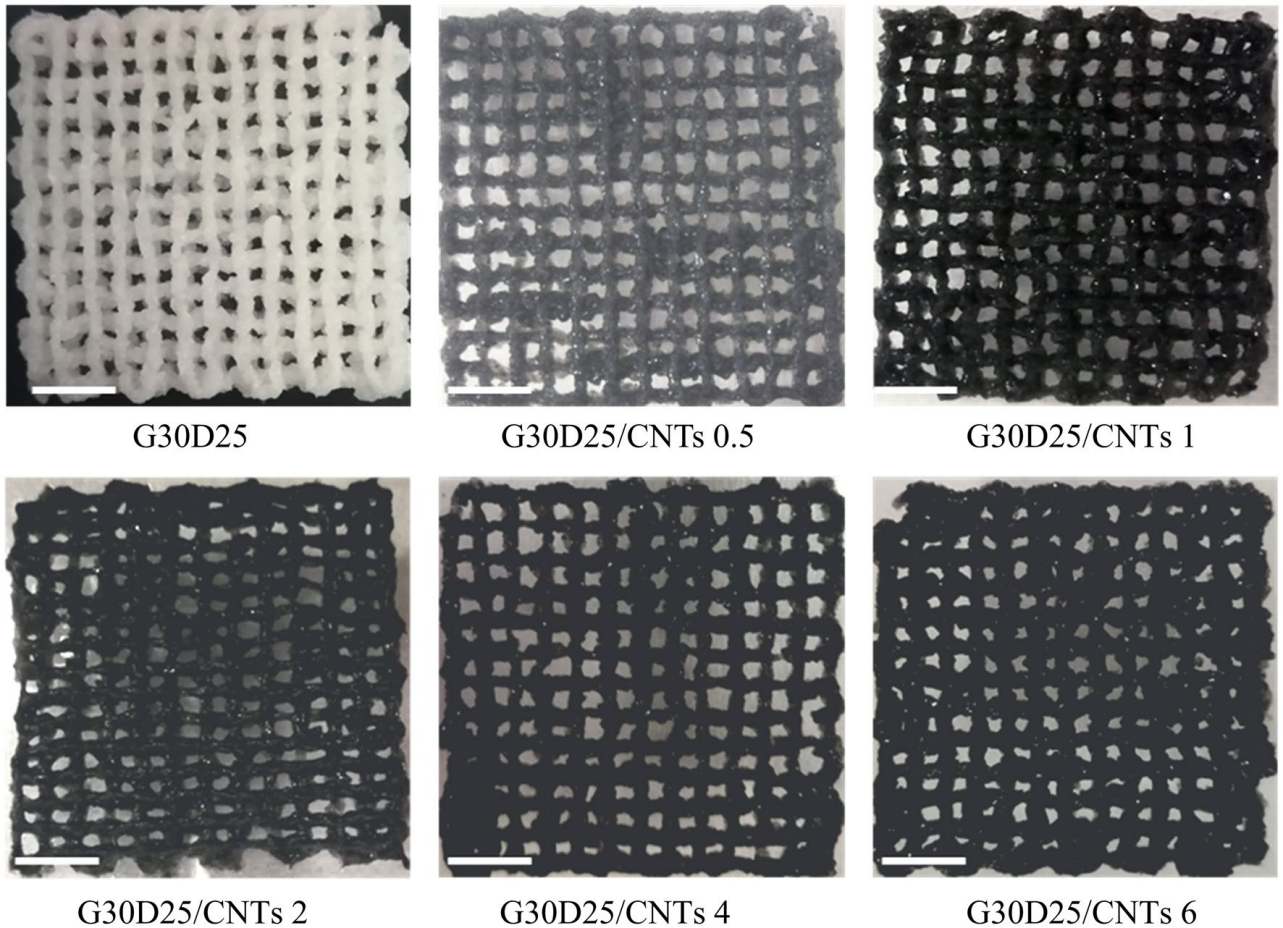
Representative macroscopic images of 3D bio-printed G30D25-CNTs scaffolds, the scale bar is 4 mm.

In order to intuitively display bio-printability of G30D25-CNTs, 3D printed grid was obtained using these electroactive hydrogels via 3D bioprinter. The specific printing process was shown in Fig. 1b. In the I step, the G30D25-CNTs hydrogel was transferred to a syringe firstly and the syringe was heat to 37 °C, the gelatin crystallites were de-crosslinked, and then extruded hydrogel through a metal needle onto the room temperature printing table. UV solidification process as the II step, the methacrylamide group of GelMA undergoes secondary crosslinking improving mechanical strength and shape fidelity. The macroscopic images and analyzed quantitatively of which were shown in Fig. 5 and Fig. S2. A dimensionless variable width index of D1//D0 was defined, where D0 is nozzle diameter and D1 is average pathwidth. The closer the value of D1/D0 is to 1, the higher the 3D printability^43^. We observed that, the scaffold using G30D25 hydrogel has severe diffusion phenomenon. Nevertheless, the printed grids using G30D25-CNTs0.5, G30D25-CNTs1, G30D25-CNTs2 and G30D25-CNTs4 all showed excellent defined structures. Therefore, the addition of carbon nanotubes could improve the bio-printability of G30D25 hydrogel. This may be due to that, when CNTs were encapsulated in G30D25 hydrogel, the shearing force during the printing process could induce the orientation of CNTs along the printing direction, which further enhanced their alignment of G30D25 hydrogel^44^. However, there are still ruptures on printed lines in the scaffold fabrication by G30D25 and G30D25-CNTs hydrogels, which was a common problem for the studies with regarding to gelatin-based materials.

### Conductivities of G30D25-CNTs electroactive scaffolds

**Figure 6:**
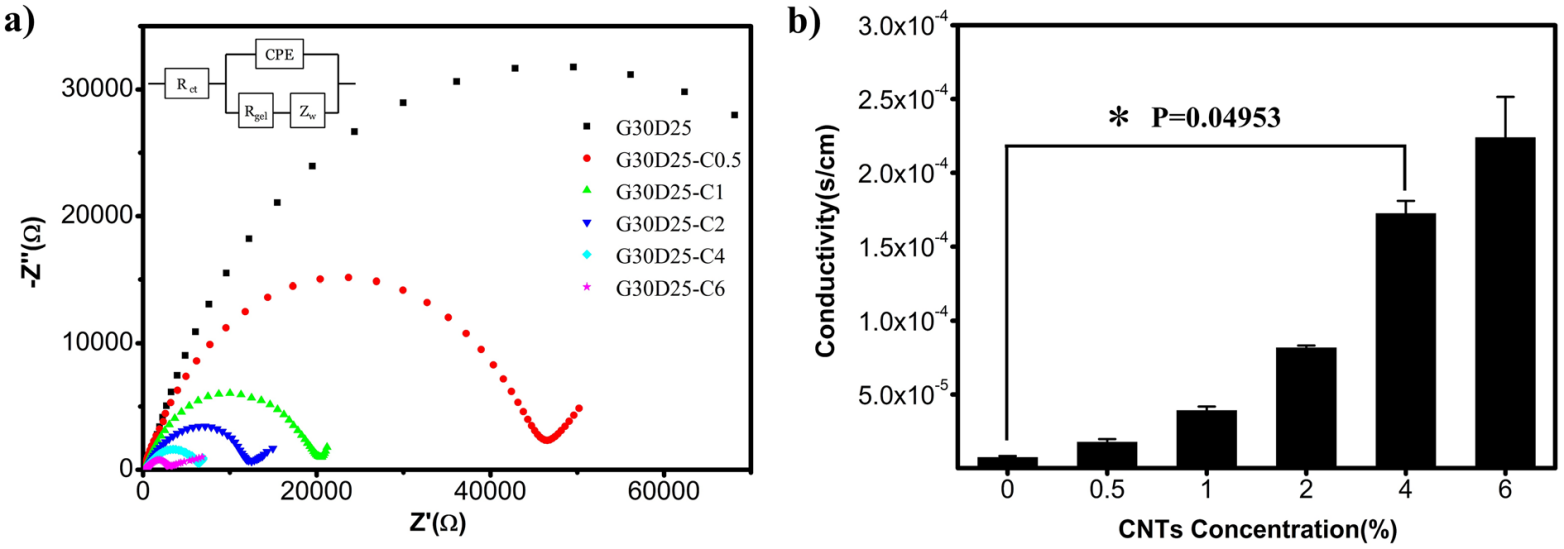
Electrical conductivities of G30D25-CNTs electroactive hydrogels scaffolds. (**a**) Nyquist plots and corresponding equivalent circuit. (**b**) Conductivity of electroactive hydrogel as a function of CNTs content. Bar graphs show mean ± S.E.M. *p < 0.05.

The conductivity of G30D25-CNTs electroactive scaffolds was determined using electrochemical impedance spectroscopy (EIS). Figure 6a displayed the Nyquist curve, which were semicircular in the low frequency region and linear in the high frequency region. The equivalent circuit of G30D25-CNTs electroactive scaffolds was analyzed using a Randles circuit, which consist of a charge transfer resistance (Rct) in series with the parallel combination of a constant phase element (CPE) and the series combination of a bulk resistance of gel (Rgel) and warburg impedance (Zw). Compared to G30D25, the semicircle diameter of G30D25-CNTs, which is equal to the electron-transfer resistance (Rct), gradually decreased with the amount of CNTs increasing from 0.5 to 6%, implying that the presence of CNTs improved the electrical conductivity. Furthermore, the electrical conductivities of G30D25-CNTs hydrogels as a function of CNTs concentration were plotted in Fig. 6b. We observed that, when CNTs content increased from 0 to 6%, the κ value enhanced from 7.41 × 10^–6^ to 2.24 × 10^–4^ s/cm, correspondingly. Only 0.5% CNTs significantly increased the conductivity of the G30D25 hydrogel, indicating that this addition has been above the threshold of a single percolation path, which was consistent with the critical percentage of single percolation (< 0.05%) reported in the literature^45^. As the concent of CNTs was above 4%, the conductivity of G30D25-CNTs hydrogel exceeded 10^–2^ S/m, which satisfied the needs of cells for micro-current stimulation. Therefore, G30D25-CNTs4 electroactive hydrogel could be further considered for application in electrical stimulation of tissue regeneration such as muscle and cardiac nerve tissue repair^36,46^.

### Wettability, swelling and degradation behavior of G30D25-CNTs hydrogels

**Figure 7:**
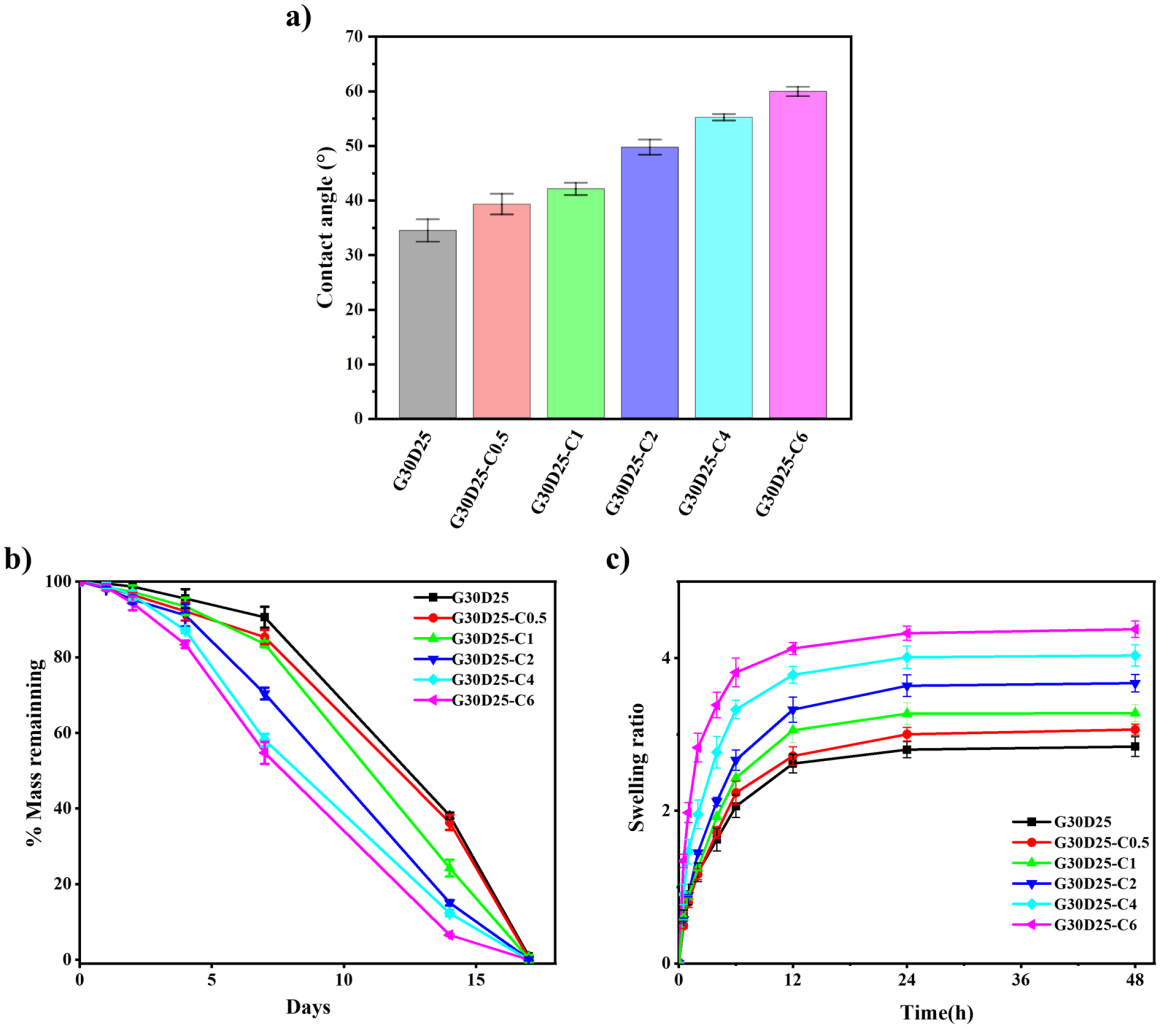
(**a**) Water contact angle values (°). Bar graphs show mean ± S.E.M; Swelling (**a**) and degradation, (**b**) curves of G30D25-CNTs hydrogels. Bar graphs show mean ± S.E.M.

Biological interactions take place at the surface of the biomaterials where they are in direct contact with the host tissue. Surfaces with a moderate wettability (contact angle of water 30–60°) have been shown to be favorable for cell adhesion and proliferation^42,47^. As shown in Fig. 7a and Fig. S3, all the G30D25-CNTs hydrogels in this study demonstrate hydrophilic surfaces. Increasing the content of CNTs from 0 to 6% results in an increase in the contact angle from 35.7° to 59.8°, indicating a reduction in surface hydrophilicity, owing to the hydrophobicity of CNTs. However, the prepared G30D25-CNTs hydrogels are still suitable for cell attachment and proliferation.

The swelling behavior of G30D25 and G30D25-CNTs hydrogels in PBS was investigated to evaluate their capacity to absorb water, and their swelling curves were shown in Fig. 7b. We observed that, the freeze-dried hydrogel swelled rapidly within 6 h and then the swelling equilibrium was reached slowly. This phenomenon can be explained that, GelMA and DF-PEG are hydrophilic polymers which contain plenty of hydrophilic groups. Therefore, water molecule could freely come into the network of the hydrogels^48^. Furthermore, with increasing CNTs content from 0 to 6%, the swelling ratio (SR) of G30D25-CNTs hydrogels increased from 2.84 to 4.38, because the micropores of G30D25-CNTs hydrogels enlarged accordingly (Fig. 4), thus the diffusition speed of water molecule accelerated^49^.

In vitro degradation of G30D25 and G30D25-CNTs hydrogels was investigated via monitoring weight loss after incubation in pH 7.4 PBS, as shown in Fig. 7c. We observed that, at the initial first two days, the rate of weight loss was slow, which may be due to that the penetration of water molecule into hydrogels was dominant at this period. Two days later, the degradation of G30D25 and G30D25-CNTs all become rapidly, owing to that, the dynamic equilibrium of Schiff base bonds inside the hydrogels was destroyed. Furthermore, with the amount of CNTs increasing, the degradation rate of G30D25-CNTs electroactive hydrogels increased correspondingly, which can be explained that the micro-pores inside the hydrogels enlarged along with the CNTs content increasing (Fig. 4), leading to the diffusion of water molecule within the networks enhanced.

### Cell toxicity assay

**Figure 8:**
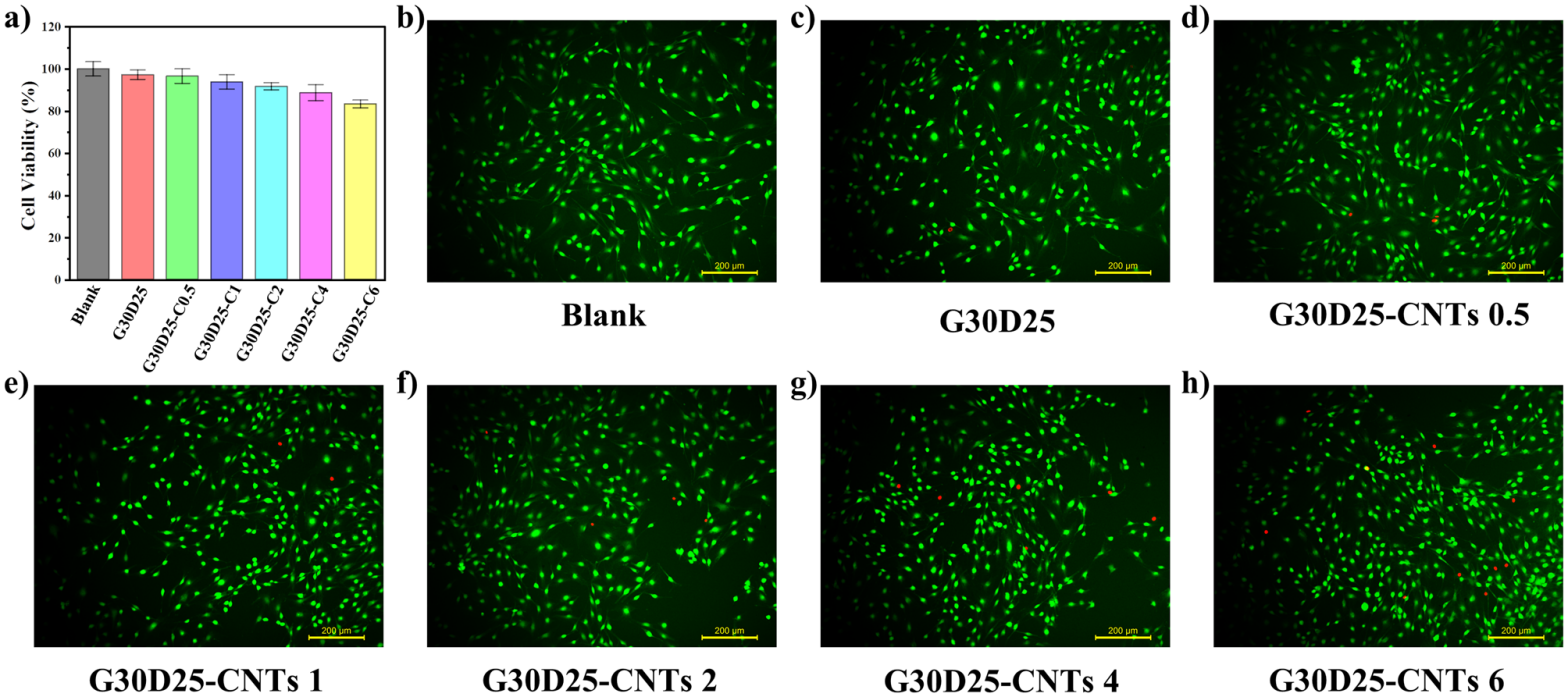
(**a**) Cell viabilities of MC3T3-E1 cells treated with extract medium from G30D25 and G30D25-CNTs hydrogels for 24 h; (**b**) confocal microscopy of Live/Dead MC3T3-E1 cells stained by Calcein-AM/PI after treating with extract liquid from G30D25 and G30D25-CNTs for 24 h.

The cytotoxicity of G30D25-CNTs hydrogels was assayed by exposing MC3T3-E1 cells to the extract medium from these hydrogels for 24 h. Potential toxic compounds can impact both cell viability (Fig. 8a) and morphology (Fig. 8b–h). In Fig. 8a, the viabilities of MC3T3-E1 cells treated with G30D25, G30D25-CNTs0.5, and G30D25-CNTs1 were 97.2%, 96.5%, and 93.8%, respectively, showing no significant difference compared to the control. The G30D25-CNTs2 and G30D25-CNTs4 groups exhibited a slight decrease in cell viability. Although the viability of the G30D25-CNTs6 group showed a noticeable decrease, it still remained above 80%. Therefore, we can conclude that the G30D25-CNTs hydrogel was non-cytotoxic^42^.

Additionally, Fig. 8b–h revealed robust green fluorescence signals in all groups, indicating intact cell morphology, with only a minimal number of dead cell nuclei stained in red. This outcome aligns with the observed trend in the cell viability test, providing further confirmation of the exceptional cell compatibility of the prepared G30D25-CNTs hydrogels.

## Conclusion

The GelMA/DF-PEG based electroactive hydrogel was prepared via schiff-base reaction and meanwhile MWCNTs were homogeneously distributed into GelMA solution. The optimized G30D25-CNTs demonstrated the shear-thinning behaviour, which ensured that they can be easily extruded and retained its plotted shape once being printed via 3D bioprinter. Moreover, we found that the conductivities of G30D25-CNTs4 and G30D25-CNTs6 hydrogels exceeded 10^–4^ S/cm, which satisfied the needs of cells for micro-current stimulation. However, compared to that of G30D25-CNTs6, the swelling behaviour and degradation ability of G30D25-CNTs4 electroactive hydrogels were more appropriate to be used as a scaffold material. Thus, the G30D25-CNTs hydrogel with 4% MWCNTs could be considered for further application in electrical stimulation of tissue regeneration such as muscle and cardiac nerve tissue repair.

### Supplementary Information

Supplementary Figures.

## Data Availability

All data analyzed during this study are included in this published article.
