# Supplementary material for: A dual-crosslinking electroactive hydrogel based on gelatin methacrylate and dibenzaldehyde-terminated telechelic polyethylene glycol for 3D bio-printing
Source: Sci Rep. 2024 Feb 19;14:4118. doi: 10.1038/s41598-024-54853-9 (PMC10876640; doi:10.1038/s41598-024-54853-9)
Supplement: Supplementary file 1 — Supplementary Figures. [file 41598_2024_54853_MOESM1_ESM.docx]

A dual-crosslinking electroactive hydrogel based on gelatin methacrylate and dibenzaldehyde-terminated telechelic polyethylene glycol for 3D bio-printing

**Yulong Wang^1,2^, Songsong Yang^1^, Heqing Cai^1^, Hailong Hu^1^, Kun Hu^1^, Zhicheng Sun^1^, Ruping Liu^1^, Yen Wei^1,3*^, Lu Han^1*^,**

^1^ The Engineering Research Center of 3D Printing and Bio-fabrication, Beijing Institute of Graphic Communication, Beijing 102600, China

^2^ School of Physical Science and Technology, Guangxi University, Nanning 530004, China

^3^ Department of Chemistry and Key Laboratory of Bioorganic Phosphorus Chemistry and Chemical Biology (Ministry of Education), Tsinghua University, Beijing 100084, China

^[*](mailto:*weiyen@tsinghua.edu.cn)^[weiyen@tsinghua.edu.cn](mailto:*weiyen@tsinghua.edu.cn), ^*^[hanlu@iccas.ac.cn](mailto:hanlu@iccas.ac.cn)


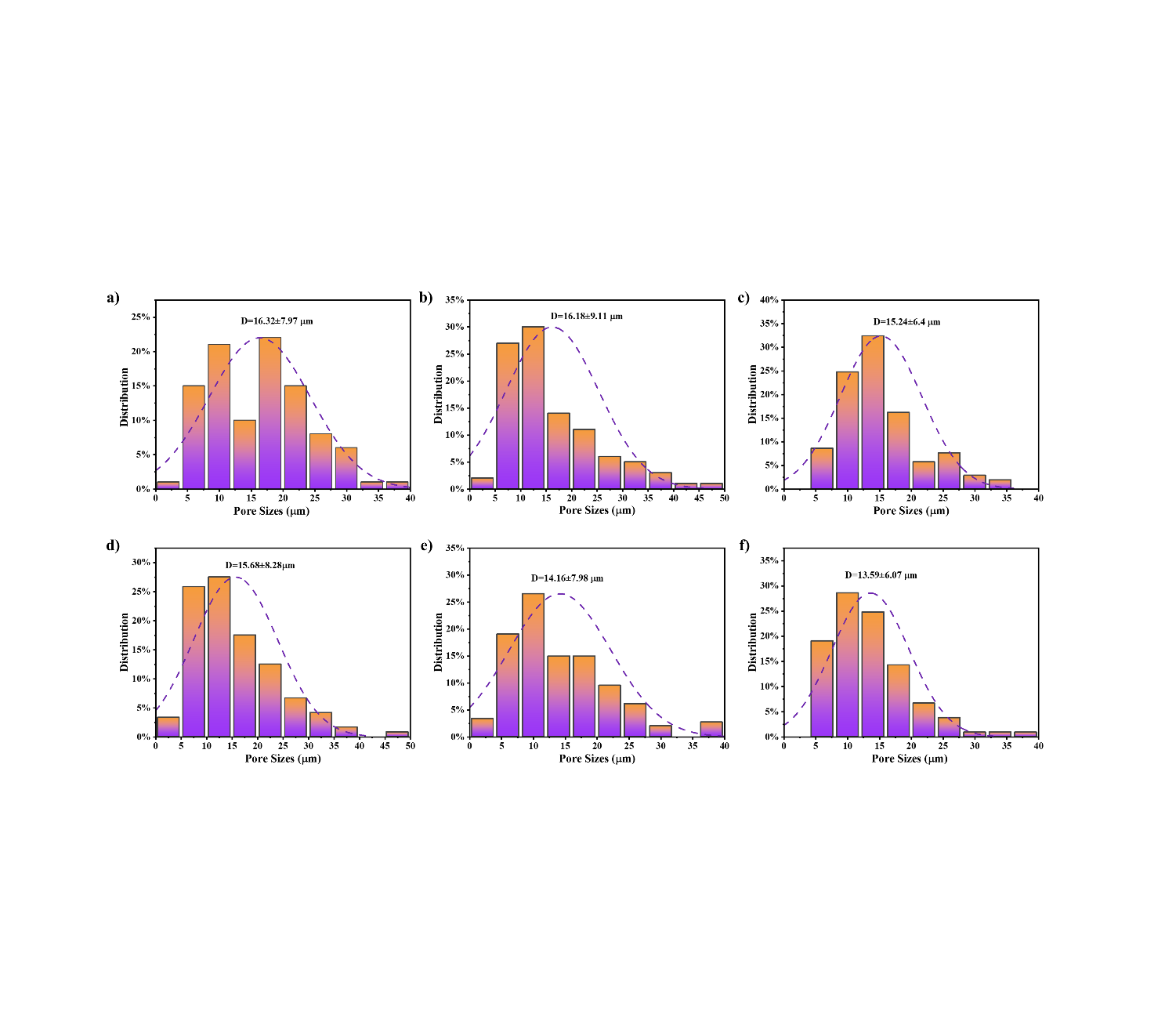


Figure S1 Quantify the pore size distribution from SEM images. a)-f) are G30D25, G30D25-CNTs0.5, G30D25-CNTs1, G30D25-CNTs2, G30D25-CNTs4 and G30D25-CNTs6 hydrogel, respectively.


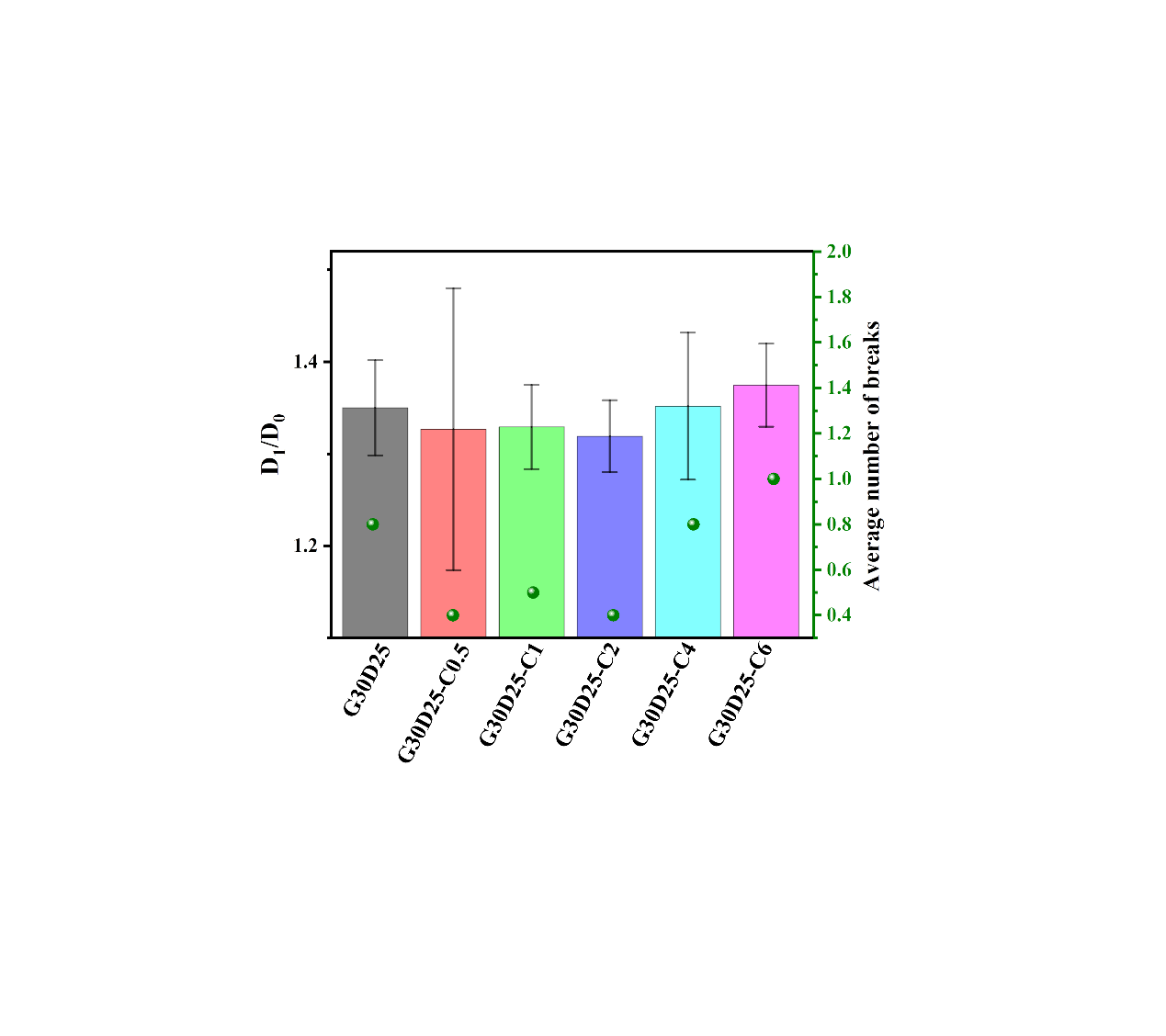


Figure S2 Quantify the ratio(D_1_/D_0_) of the true diameter of the printed line to the set value, and the average number of breaks.

Herein, Photoshop was used to isolate the continuous printed lines of different G30D25-CNTs groups. A dimensionless variable width index of D1//D0 was defined, where D0 is nozzle diameter and D1 is average pathwidth. The closer the value of D1/D0 is to 1, the higher the 3D printability[1]. Furthermore, the average number of line breaks serves as an evaluation metric. It is defined as the ratio of the total number of breaks to the overall number of lines statistically. This metric reflects the printing continuity, with a smaller value indicating enhanced printability.


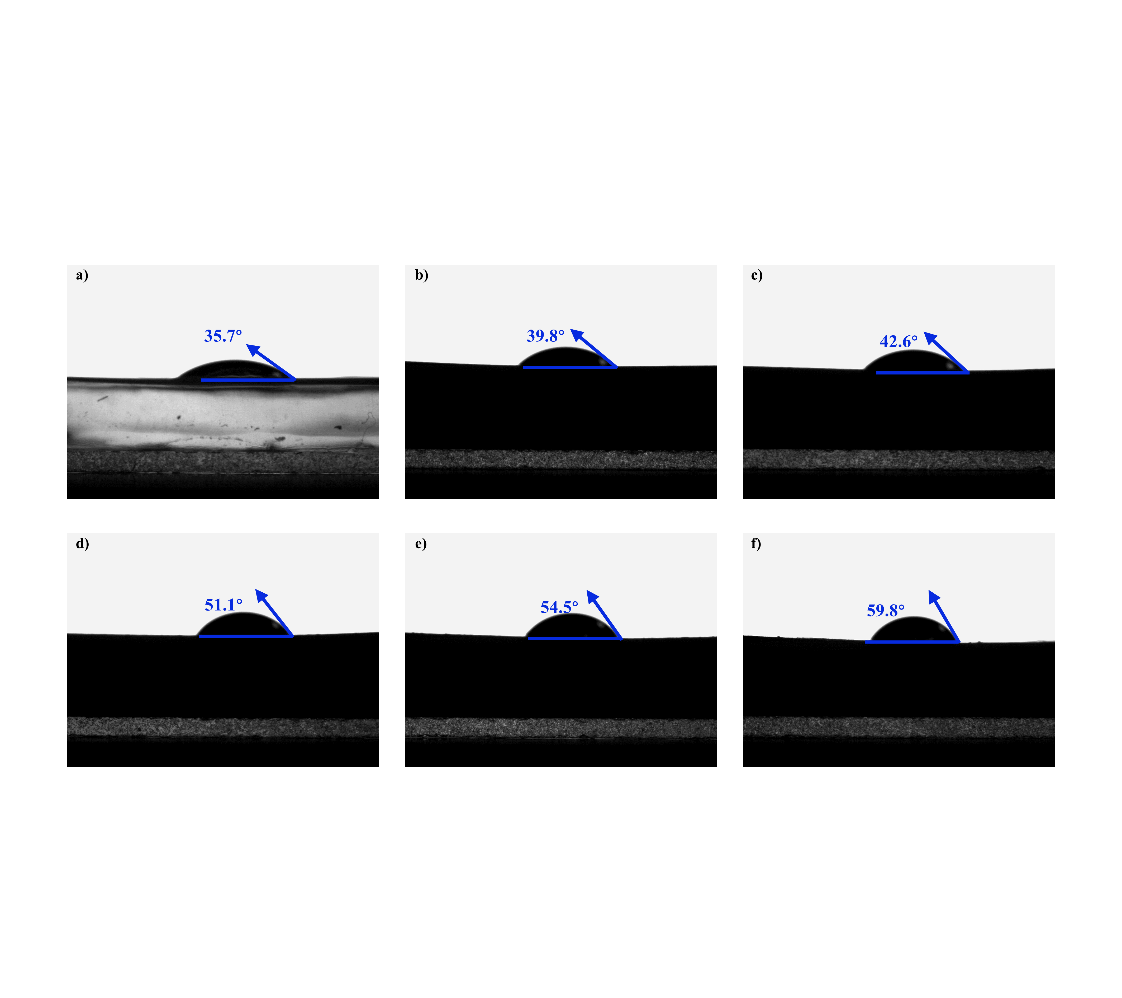


Figure S3 a)-f) Water contact angle of the a) G30D25-CNTs, b) G30D25-CNTs0.5, c) G30D25-CNTs1, d) G30D25-CNTs2, e) G30D25-CNTs4 and f) G30D25-CNTs6 hydrogel.


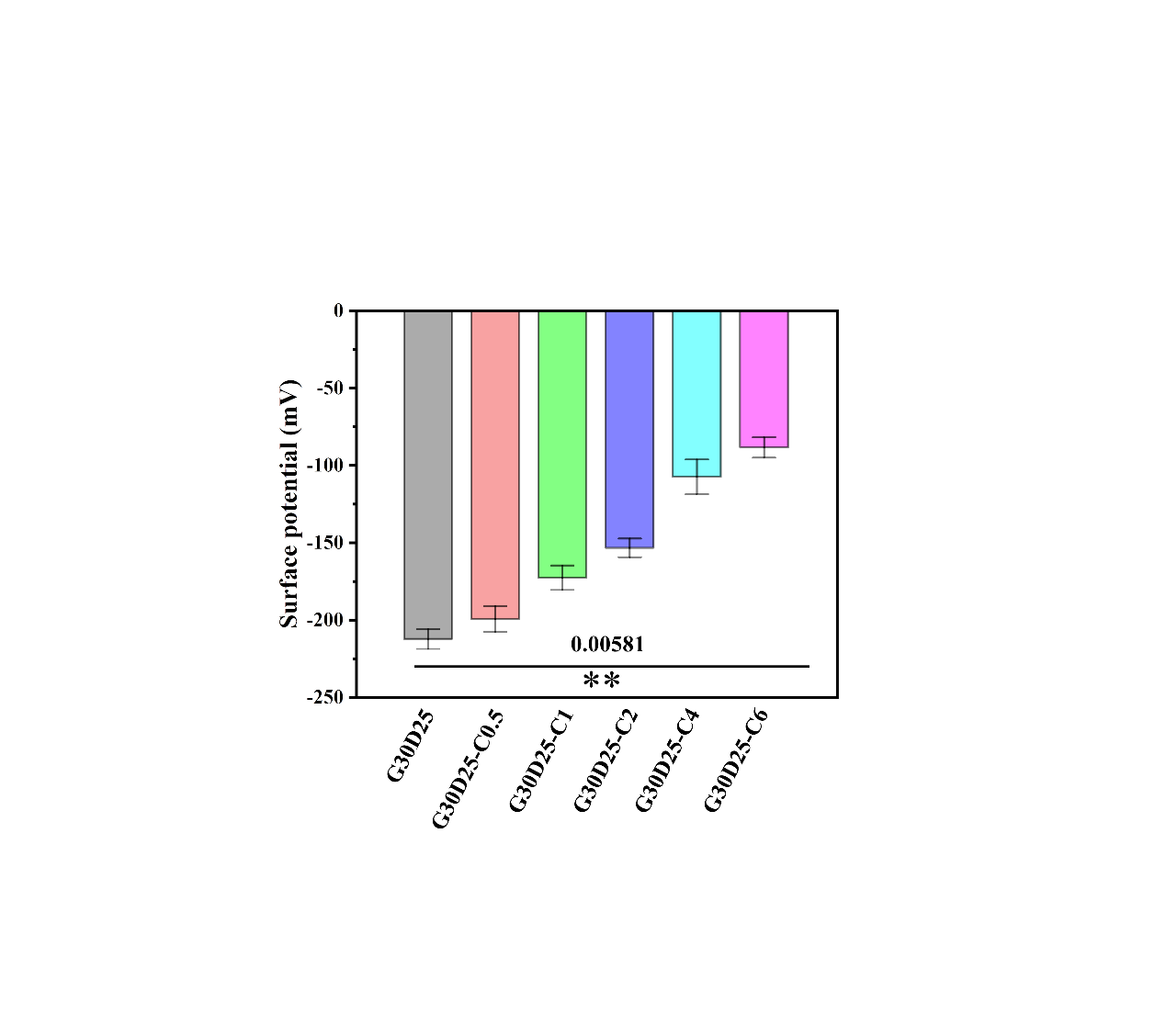


Figure S4 a)-f) surface potential of the a) G30D25, b) G30D25-CNTs0.5, c) G30D25-CNTs1, d) G30D25-CNTs2, e) G30D25-CNTs4 and f) G30D25-CNTs6 hydrogel.

The surface potential of G30D25-CNTs hydrogel was detected using an electrostatic field meter (FMX-003, SMICO). We can see that, the surfaces of all the G30D25-CNTs hydrogels exhibit negative charge, and this negative charge decreases gradually with increasing carbon nanotube content.

[1] K.H. Kang, L.A. Hockaday, J.T. Butcher, Quantitative optimization of solid freeform deposition of aqueous hydrogels, Biofabrication 5(3) (2013).
